# Lower peripheral T- and NK-cell counts are associated with systemic inflammation and nutritional status in hospitalized gastrointestinal surgery patients

**DOI:** 10.3389/fmed.2026.1916752

**Published:** 2026-10-21

**Authors:** Zhi Duan, Xiangjuan Jiao, Ning Wang, Wubin Zhou, Jiao He, Lingyong Liu, Qi Zuo, Zhen Li

**Affiliations:** 1 Department of Pathology, The First Hospital of Changsha/The Affiliated Changsha Hospital of Xiangya School of Medicine, Central South University, Changsha, Hunan, China; 2 Changsha High-Throughput Sequencing Technology Innovation Center for Gastrointestinal Tumor Precision Medicine, Changsha, Hunan, China; 3 Department of Infectious and Immunology, The First Hospital of Changsha/The Affiliated Changsha Hospital of Xiangya School of Medicine, Central South University, Changsha, Hunan, China; 4 Department of Information and Data, The First Hospital of Changsha/The Affiliated Changsha Hospital of Xiangya School of Medicine, Central South University, Changsha, Hunan, China; 5 Department of Emergency, The First Hospital of Changsha/The Affiliated Changsha Hospital of Xiangya School of Medicine, Central South University, Changsha, Hunan, China

**Keywords:** gastrointestinal surgery, inflammation, lymphocyte subsets, natural killer cells, nutritional status, T lymphocytes

## Abstract

**Background:**

Systemic inflammation and impaired nutritional status may be associated with altered cellular immune profiles in surgical inpatients. However, peripheral T- and NK-cell profiles and their clinical correlates remain insufficiently characterized in hospitalized gastrointestinal surgery patients.

**Methods:**

This retrospective observational study included hospitalized patients from a gastrointestinal surgery department who underwent peripheral blood lymphocyte subset testing between January 2024 and May 2025. Patients were classified into disease categories according to discharge diagnoses. T lymphocytes, natural killer (NK) cells, B lymphocytes, and NKT cells were analyzed together with inflammatory markers, including interleukin-6 (IL-6), C-reactive protein (CRP), white blood cell count, neutrophil-to-lymphocyte ratio (NLR), lymphocyte-to-monocyte ratio (LMR), and nutritional markers including serum albumin and hemoglobin. Group comparisons, correlation analyses, and multivariable regression were performed. Continuous T-cell and NK-cell counts were the primary outcomes; cohort-defined low-count status was a secondary exploratory outcome. Sensitivity analyses included multiple imputation and Firth penalized logistic regression.

**Results:**

After excluding one patient with missing diagnosis, 887 patients were included, of whom 623 had complete data for multivariable analysis. Higher neutrophil-to-lymphocyte ratio (NLR) was associated with lower continuous T-cell and NK-cell counts and higher odds of low-count status across complete-case and sensitivity analyses. In models pooled across 50 imputed datasets, obstruction/perforation-related acute abdomen, compared with benign anorectal diseases, was associated with lower T-cell counts (adjusted geometric mean ratio [aGMR] = 0.822, 95% CI 0.706–0.958, *P* = 0.012) and lower NK-cell counts (aGMR = 0.821, 95% CI 0.677–0.995, *P* = 0.045). These associations did not reach statistical significance in the complete-case models, indicating sensitivity to missing-data handling. Albumin associations varied by outcome and analysis.

**Conclusion:**

The primary findings concern associations of continuous T-cell and NK-cell counts with inflammatory and nutritional markers, with NLR showing consistent associations across analyses. Cohort-defined low-count status was a secondary, exploratory outcome. These findings do not establish causality or validated clinical thresholds and require prospective validation with standardized sampling and perioperative data.

## Introduction

Inflammation is a central biological process in gastrointestinal surgical diseases. Acute appendicitis, intra-abdominal infection, obstruction, perforation, inflammatory intestinal disorders, and gastrointestinal malignancies all involve complex interactions between tissue injury, microbial or tumor-associated stimuli, innate immune activation, and adaptive immune responses (1). In clinical practice, inflammatory status is commonly assessed using markers such as white blood cell count, CRP, IL-6, procalcitonin, and composite indices such as NLR. These markers are useful, but they provide only an indirect view of immune cell distribution and do not fully capture the cellular immune profile of the patient.

Peripheral blood lymphocyte subsets, including T lymphocytes, NK cells, B lymphocytes, and NKT cells, are increasingly measured in hospitalized patients (2). T lymphocytes are key effectors of adaptive cellular immunity, NK cells participate in early antiviral and antitumor responses, B lymphocytes reflect humoral immune status, and NKT cells bridge innate and adaptive immunity (3, 4). Alterations in these subsets may reflect acute stress, systemic inflammation, immune exhaustion, nutritional compromise, or disease-specific immune remodeling (5, 6). However, in gastrointestinal surgery, lymphocyte subset testing is often interpreted case by case rather than integrated with routine inflammatory and nutritional markers across diagnostic groups.

Nutritional status is also tightly linked to immune competence (7, 8). Serum albumin and hemoglobin are not pure nutritional markers, but in hospitalized surgical patients they are clinically meaningful indicators of systemic illness, protein reserve, inflammation, and perioperative risk (9). Malnutrition and hypoalbuminemia may contribute to impaired cellular immunity, while inflammation itself may suppress albumin synthesis and redistribute circulating lymphocyte populations. Therefore, simultaneous analysis of lymphocyte subsets, inflammatory burden, and nutritional markers may provide a more comprehensive picture of host status in gastrointestinal surgical patients (10).

The present retrospective study aimed to characterize flow cytometry-based peripheral lymphocyte subset profiles and to identify clinical correlates of lower circulating T-cell and NK-cell counts in hospitalized gastrointestinal surgery patients. We hypothesized that systemic inflammatory burden and impaired nutritional status would be independently associated with lower circulating cellular immune counts.

## Methods

### Study design and patients

This was a single-center retrospective observational study conducted in the Department of Gastrointestinal Surgery at the First Hospital of Changsha. Consecutive hospitalized patients who underwent peripheral blood lymphocyte subset testing during the study period were screened. The study period was defined according to admission dates as January 2024 to May 2025.

The inclusion criteria were: (1) hospitalization in the gastrointestinal surgery department; (2) available peripheral blood lymphocyte subset test results; (3) available discharge diagnosis; and (4) available routine laboratory data near the lymphocyte subset test date. The exclusion criteria were: (1) missing discharge diagnosis; (2) missing key lymphocyte subset variables; or (3) duplicated records without a reliable matching date. When multiple laboratory records were available, results were matched by patient name, hospitalization number, and the closest test date, with date precision to the day.

### Disease classification

Discharge diagnoses were reviewed and automatically grouped into clinically meaningful disease categories. The final categories were acute infection/inflammation, gastrointestinal tumors, obstruction/perforation-related acute abdomen, benign anorectal diseases, hernia and abdominal wall diseases, benign polyps/diverticular and benign intestinal diseases, stoma-related postoperative conditions, and other diseases. Benign anorectal diseases were used as the reference category in regression models because they represented a relatively low-inflammatory surgical disease group in this dataset.

### Laboratory variables

Peripheral blood lymphocyte subset variables included percentages and absolute counts of T lymphocytes, NK cells, B lymphocytes, and NKT cells. The primary immune outcomes were absolute T-cell and NK-cell counts because they more directly reflect the circulating cellular immune pool than percentages alone. Inflammatory and hematologic markers included IL-6, CRP, procalcitonin, white blood cell count, neutrophil count, lymphocyte count, platelet count, NLR, LMR, and platelet-to-lymphocyte ratio (PLR). Nutritional and metabolic markers included serum albumin, total protein, hemoglobin, red blood cell count, total cholesterol, triglycerides, and other routine biochemical parameters.

### Flow cytometric TBNK assay

Peripheral blood lymphocyte subsets were measured by flow cytometry according to the institutional standard operating procedure of the Department of Pathology. Peripheral venous blood (2 mL) was collected into EDTA anticoagulant tubes and analyzed using a BEAM DIAG flow cytometer (BeamCyte-1026). Samples were gently inverted to ensure adequate anticoagulation, stored at room temperature (18 °C–25 °C), protected from freezing or excessive heat, and analyzed within 24 h after collection. For each test, 20 μL of fluorochrome-conjugated antibody cocktail was added to a labeled flow cytometry tube, followed by 50 μL of well-mixed anticoagulated whole blood using a reverse pipetting method. Two separate antibody panels were used: CD3-FITC/CD8-PE/CD45-PerCP-Cy5.5/CD4-APC for identifying total T cells and CD4+ and CD8+ T-cell subsets, and CD3-FITC/CD16/CD56-PE/CD45-PerCP-Cy5.5/CD19-APC for identifying T cells, B cells, NK cells, and CD3 + CD16/CD56 + populations. T cells were defined as CD3 + lymphocytes, B cells as CD19 + lymphocytes, NK cells as CD3 − CD16/CD56 + lymphocytes, and NKT cells as CD3 + CD16/CD56 + lymphocytes. After vortex mixing, samples were incubated for 15 min at room temperature in the dark. Subsequently, 450 μL of freshly prepared 1× lysing solution was added, mixed thoroughly, and incubated for 10 min in the dark to lyse erythrocytes. Before acquisition, samples were mixed again to minimize cell sedimentation. Flow cytometric data were acquired within 24 h using Cytosys software, and lymphocyte subset percentages and absolute counts were generated according to the laboratory analysis template. Samples with hemolysis, lipemia, jaundice, abnormal scatter patterns, or suspected technical problems were reviewed and rechecked when necessary. The flow cytometry gating strategy is shown in Supplementary Figure S1.

### Statistical analysis

All statistical analyses were performed using Python version 3.12.13. Continuous variables were summarized as medians and interquartile ranges because most laboratory variables were non-normally distributed. Categorical variables were summarized as counts and percentages when available. Comparisons among disease categories were performed using Kruskal–Wallis tests for continuous variables and chi-square tests or Fisher's exact tests for categorical variables, as appropriate. When overall comparisons were significant, pairwise Wilcoxon rank-sum tests were performed using benign anorectal diseases as the reference group, with Bonferroni adjustment within each variable. Spearman correlation analyses were used to evaluate associations between lymphocyte subset counts and inflammatory or nutritional markers.

Multivariable linear regression models were fitted for log-transformed T-cell and NK-cell absolute counts. The main independent variable was disease category, with adjustment for sex (male vs. female), age per 10 years, log1p-transformed IL-6, log1p-transformed CRP, albumin per 5 g/L, and log1p-transformed NLR. Results were exponentiated and reported as adjusted geometric mean ratios (aGMRs, equivalent to exp[beta]), where values below 1.0 indicate lower expected lymphocyte subset counts. Logistic regression models were also fitted for low T-cell and low NK-cell counts, defined as values in the lowest quartile of the study cohort. Continuous log-transformed T-cell and NK-cell counts were the primary outcomes, whereas cohort-defined low-count outcomes were secondary exploratory analyses. Low-count status was defined as a value at or below the cohort-specific 25th percentile. These thresholds were not intended to diagnose clinical lymphopenia. Results were reported as odds ratios (ORs) with 95% confidence intervals (CIs). A two-sided *P* value <0.05 was considered statistically significant. Analyses were performed using custom scripts based on the cleaned analysis dataset. Laboratory values recorded as zero for CRP, IL-6, or NLR were treated as missing when they were judged to reflect unreported or unperformed tests rather than true biological zero values. Because the complete-case analysis excluded 264 of 887 patients, we performed multiple imputation by chained equations under a missing-at-random assumption conditional on the included variables. IL-6, CRP, albumin, and NLR were imputed using Gaussian parameter draws and predictive mean matching with 20 candidate donors. The imputation models included disease category, sex, age, log-transformed T-cell and NK-cell counts, both cohort-defined low-count indicators, and the other imputed covariates. IL-6, CRP, and NLR were imputed on the log1p scale and albumin on the per-5-g/L scale. After 20 burn-in cycles, 50 completed datasets were retained at five-cycle intervals. Estimates were combined using Rubin's rules, with Barnard-Rubin degrees of freedom for *t*-based confidence intervals and tests. Imputed values were checked against observed donor values, and an independent-seed run was used to assess simulation stability. Firth penalized logistic regression was additionally performed on complete cases; its confidence intervals and *P* values used an expected-information Wald approximation. These sensitivity analyses were exploratory. In an exploratory analysis, the original obstruction/perforation-related acute-abdomen category was divided using discharge-diagnosis keywords. Diagnoses containing perforation, rupture, peritonitis, sepsis, or septic shock were assigned to the perforation/peritonitis subgroup, with precedence over obstruction-related keywords. Remaining diagnoses containing obstruction, intussusception, incarceration, stenosis, or volvulus were assigned to the obstruction/incarceration subgroup. In this dataset, all 23 patients assigned to perforation/peritonitis had explicit perforation or peritonitis keywords, and no patients remained unmatched. This text-based classification was not independently adjudicated against operative findings.

## Results

### Study population and disease categories

A total of 888 records were available in the cleaned analysis dataset. One record with missing diagnosis was excluded, leaving 887 observations for the final analysis. The disease categories were acute infection/inflammation (*n* = 258), gastrointestinal tumors (*n* = 150), obstruction/perforation-related acute abdomen (*n* = 134), benign polyps/diverticular and benign intestinal diseases (*n* = 136), benign anorectal diseases (*n* = 74), hernia and abdominal wall diseases (*n* = 32), stoma-related postoperative conditions (*n* = 16), and other diseases (*n* = 87). Age and length of stay differed significantly across disease categories (both *P* < 0.001). Patients with gastrointestinal tumors were older and had the longest median hospital stay (18.00 days, IQR 12.75–22.00) (Table 1).

**Table 1:** Baseline characteristics according to disease category.

Variable	*P*	INF	TUM	OPA	ANO	HER	POL	STO	OTH
*n*		258	150	134	74	32	136	16	87
Male sex, *n* (%)	0.001	131 (50.8%)	94 (62.7%)	68 (50.7%)	48 (64.9%)	28 (87.5%)	80 (58.8%)	12 (75.0%)	51 (58.6%)
Age, years	<0.001	42.00 (30.25–59.75); *n* = 258	66.00 (60.00–72.00); *n* = 150	64.50 (52.00–72.00); *n* = 134	49.00 (34.25–63.75); *n* = 74	69.50 (60.00–75.00); *n* = 32	61.00 (53.00–71.00); *n* = 136	68.50 (60.25–73.75); *n* = 16	55.00 (39.50–69.00); *n* = 87
LOS, days	<0.001	6.80 (5.50–9.10); *n* = 227	18.00 (12.75–22.00); *n* = 144	7.60 (4.90–12.88); *n* = 134	7.90 (6.22–10.00); *n* = 70	8.00 (5.00–12.45); *n* = 31	6.70 (4.80–9.25); *n* = 135	14.00 (7.67–19.85); *n* = 16	7.90 (5.05–11.80); *n* = 87

Continuous variables are presented as median (interquartile range). Sex was complete for all included patients. LOS, length of stay; LOS data were available for 844 of 887 patients; the *n* shown for each group indicates available values. INF, acute infection/inflammation; TUM, GI tumors; OPA, obstruction/perforation acute abdomen; ANO, benign anorectal diseases; HER, hernia/abdominal wall diseases; POL, benign polyps/diverticular disease; STO, stoma-related conditions; OTH, other diseases.

### Disease categories showed distinct lymphocyte subset profiles

Absolute lymphocyte subset counts differed significantly across disease categories. Median T-cell counts were lowest in obstruction/perforation-related acute abdomen (492 cells/μL, IQR 309–764), followed by acute infection/inflammation (652 cells/μL, IQR 477–951). In contrast, higher T-cell counts were observed in benign anorectal diseases (854 cells/μL, IQR 647–1,184) and benign polyps/diverticular and benign intestinal diseases (936 cells/μL, IQR 685–1,146). NK-cell counts also differed among categories (*P* < 0.001), with low values in obstruction/perforation-related acute abdomen (110 cells/μL, IQR 70.3–175) and acute infection/inflammation (124 cells/μL, IQR 80.5–178), whereas gastrointestinal tumors showed a relatively higher median NK-cell count (175 cells/μL, IQR 117–274) (Figure 1; Supplementary Table S1). B-cell and NKT-cell measures also showed significant group differences (Supplementary Table S1).

**Figure 1:**
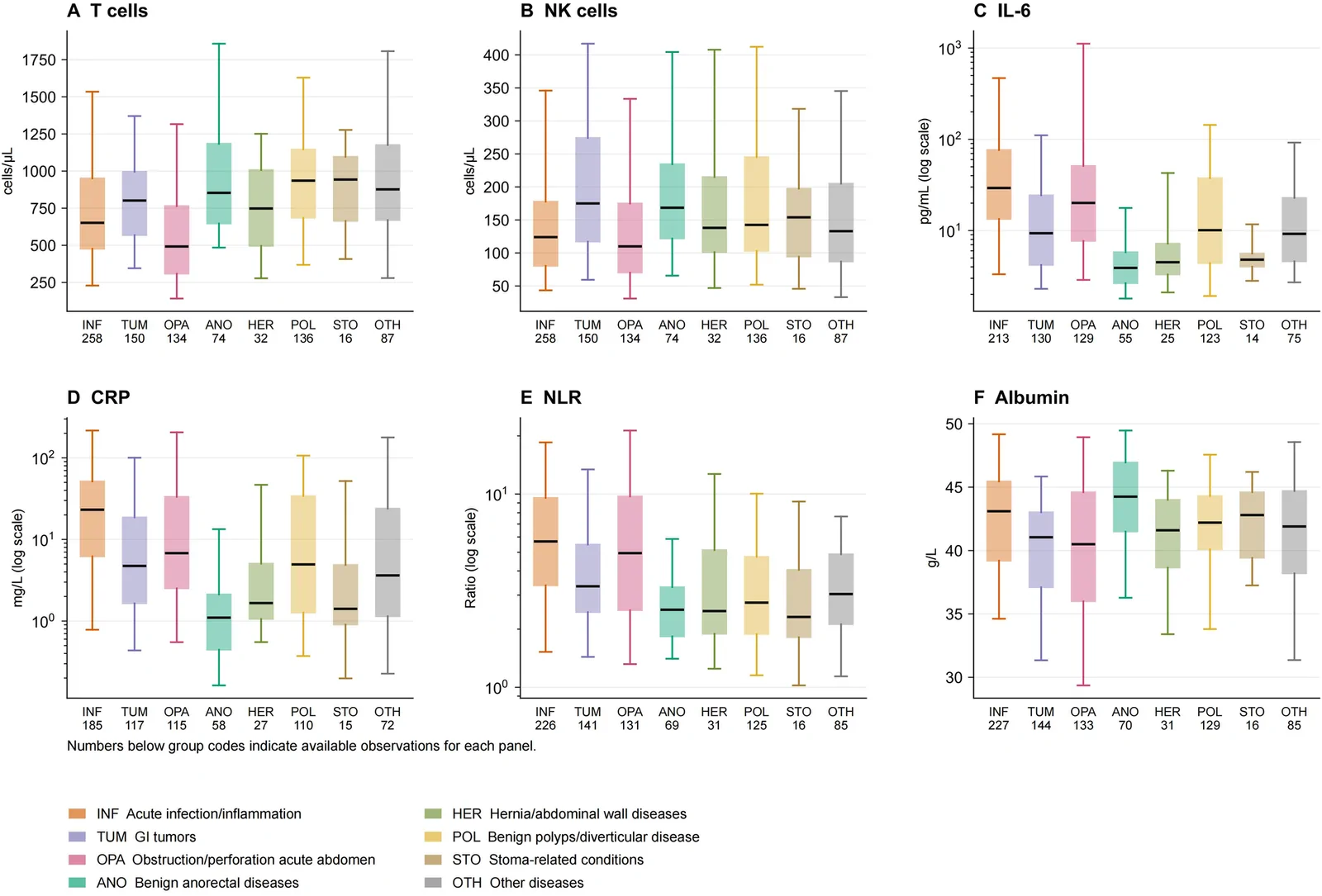
Immune, inflammatory, and nutritional profiles by disease category. Boxplots show **(A)** T-cell count, **(B)** NK-cell count, **(C)** IL-6, **(D)** CRP, **(E)** NLR, and **(F)** serum albumin across disease categories. Boxes represent medians and interquartile ranges, and whiskers represent the 5th to 95th percentiles. IL-6, CRP, and NLR are displayed on log scales. Disease categories are shown using abbreviated labels and defined in the figure legend.

Compared with benign anorectal diseases, T-cell counts were significantly lower in acute infection/inflammation and obstruction/perforation-related acute abdomen. These findings suggest that acute inflammatory surgical conditions showed descriptively lower circulating T-cell counts, particularly in patients with obstruction or perforation-related acute abdominal disease (Supplementary Table S2).

### Inflammatory burden and nutritional markers differed across disease categories

Inflammatory markers were highest in acute inflammatory categories. IL-6 differed significantly across groups (*P* < 0.001), with median values of 29.30 pg/mL (IQR 13.40–76.40) in acute infection/inflammation and 20.10 pg/mL (IQR 7.70–51.10) in obstruction/perforation-related acute abdomen, compared with 3.90 pg/mL (IQR 2.65–5.80) in benign anorectal diseases. CRP and NLR showed similar patterns. Median NLR was 5.68 (IQR 3.37–9.56) in acute infection/inflammation and 4.95 (IQR 2.50–9.72) in obstruction/perforation-related acute abdomen, compared with 2.52 (IQR 1.83–3.30) in benign anorectal diseases (Figure 1; Supplementary Table S1).

LMR was lower in the more inflammatory categories, with median values of 1.90 (IQR 1.35–2.86) in acute infection/inflammation and 2.05 (IQR 1.24–3.48) in obstruction/perforation-related acute abdomen, compared with 3.59 (IQR 2.91–4.49) in benign anorectal diseases (Supplementary Table S1). Albumin also differed across groups (*P* < 0.001), with lower median levels in obstruction/perforation-related acute abdomen (40.50 g/L, IQR 36.00–44.60) and gastrointestinal tumors (41.05 g/L, IQR 37.10–43.02) than in benign anorectal diseases (44.25 g/L, IQR 41.50–46.95) (Figure 1; Supplementary Table S1).

### Associations between lymphocyte subsets, inflammation, and nutrition

Spearman correlation analyses showed that T-cell count was inversely correlated with IL-6 (*r* = −0.336, *n* = 764), CRP (*r* = −0.233, *n* = 699), and NLR (*r* = −0.487, *n* = 824), and positively correlated with LMR (*r* = 0.487, *n* = 823) and albumin (*r* = 0.200, *n* = 835). NK-cell count showed similar but weaker associations, including inverse correlations with IL-6 (*r* = −0.186, *n* = 764), CRP (*r* = −0.199, *n* = 699), and NLR (*r* = −0.262, *n* = 824), and a positive correlation with LMR (*r* = 0.246, *n* = 823). All reported correlations remained statistically significant after Benjamini–Hochberg FDR adjustment (Figure 2; Supplementary Table S3).

**Figure 2:**
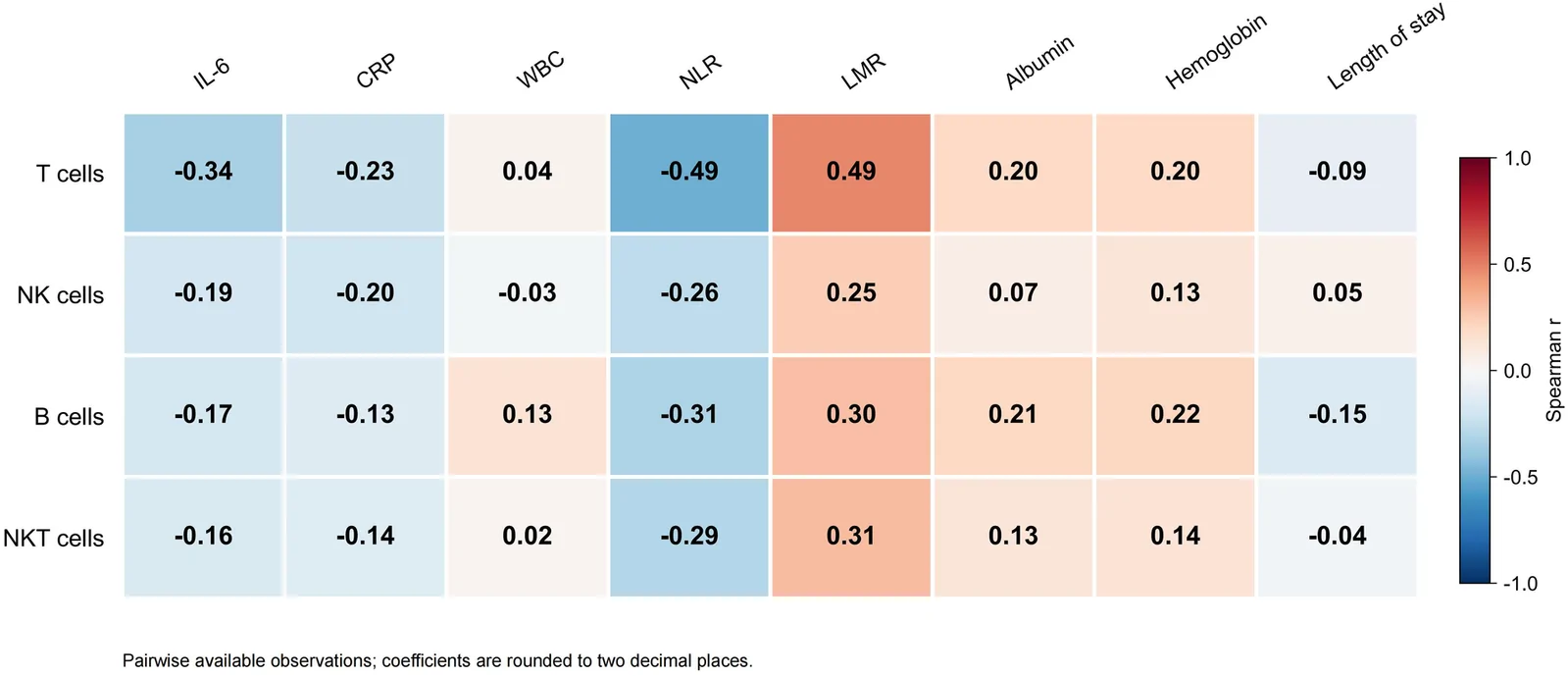
Correlations between lymphocyte subsets and clinical markers. Heatmap showing Spearman correlation coefficients between peripheral lymphocyte subset counts and inflammatory, nutritional, and clinical markers, calculated using pairwise available observations. Blue indicates negative correlations and red indicates positive correlations. Coefficients are rounded to two decimal places. Sample sizes, raw *P* values, and false discovery rate-adjusted *P* values are provided in Supplementary Table S3.

### Multivariable analysis

The complete-case multivariable models included 623 patients with complete data for disease category, sex, age, IL-6, CRP, albumin, NLR, and the relevant lymphocyte outcome. Patients with incomplete covariate data (*n* = 264) were compared with complete-case patients to assess potential selection bias (Supplementary Table S4). Sex was included as a covariate in all adjusted models. Variance inflation factors were calculated to assess multicollinearity; the maximum VIF was 4.141. Spearman correlation *P* values were adjusted using the Benjamini–Hochberg false discovery rate method. Pairwise comparisons vs. benign anorectal diseases were Bonferroni adjusted within each variable (Supplementary Table S2).

For log-transformed T-cell count, obstruction/perforation-related acute abdomen was not significantly associated after adjustment (aGMR = 0.867, 95% CI 0.714–1.052, *P* = 0.148), whereas higher NLR was associated with lower T-cell count (aGMR = 0.712, 95% CI 0.659–0.769, *P* < 0.001) and higher albumin was associated with higher T-cell count (aGMR = 1.056 per 5 g/L, 95% CI 1.006–1.108, *P* = 0.027). For log-transformed NK-cell count, the association with obstruction/perforation-related acute abdomen was attenuated and did not reach statistical significance after adjustment for sex and other covariates (aGMR = 0.810, 95% CI 0.638–1.029, *P* = 0.084). Higher NLR was independently associated with lower NK-cell count (aGMR = 0.719, 95% CI 0.654–0.791, *P* < 0.001), while higher albumin was associated with higher NK-cell count (aGMR = 1.072 per 5 g/L, 95% CI 1.010–1.138, *P* = 0.022) (Table 2; Figure 3; Supplementary Table S5).

**Table 2:** Core multivariable regression results from complete-case analyses (*n* = 623).

Model	Predictor	Effect estimate	95% CI	*P*
Linear: log(T count)	Obstruction/perforation vs. benign anorectal	0.867	0.714–1.052	0.148
	Male vs. female	0.959	0.879–1.046	0.342
	Age per 10 years	0.958	0.932–0.984	0.002
	Albumin, per 5 g/L	1.056	1.006–1.108	0.027
	NLR, log1p transformed	0.712	0.659–0.769	<0.001
Linear: log(NK count)	Obstruction/perforation vs. benign anorectal	0.810	0.638–1.029	0.084
	Male vs. female	1.055	0.948–1.174	0.325
	Age per 10 years	1.088	1.053–1.125	<0.001
	Albumin, per 5 g/L	1.072	1.010–1.138	0.022
	NLR, log1p transformed	0.719	0.654–0.791	<0.001
Logistic: low T count	Obstruction/perforation vs. benign anorectal	2.939	1.001–8.627	0.050
	Male vs. female	1.244	0.799–1.937	0.333
	Age per 10 years	1.140	0.994–1.307	0.061
	Albumin, per 5 g/L	0.809	0.642–1.018	0.070
	NLR, log1p transformed	3.077	2.075–4.563	<0.001
Logistic: low NK count	Obstruction/perforation vs. benign anorectal	1.026	0.424–2.482	0.955
	Male vs. female	0.866	0.582–1.290	0.481
	Age per 10 years	0.785	0.694–0.888	<0.001
	Albumin, per 5 g/L	0.702	0.567–0.869	0.001
	NLR, log1p transformed	2.112	1.487–2.999	<0.001

Linear models report adjusted geometric mean ratios (aGMRs, equivalent to exp[beta]); logistic models report odds ratios. Full models are provided in Supplementary Table S5.

**Figure 3:**
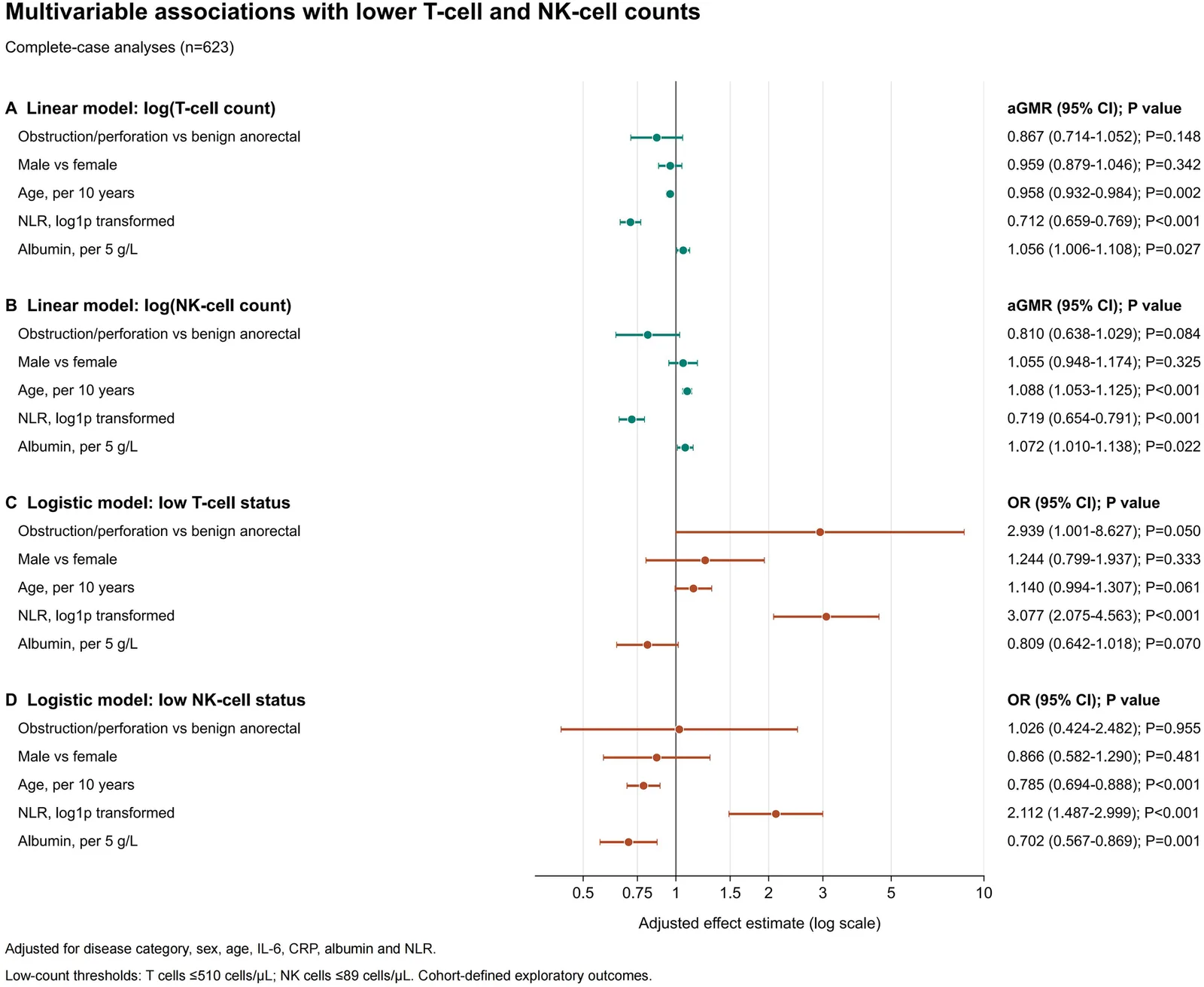
Multivariable associations with lower T-cell and NK-cell counts. Panels show results for **(A)** log-transformed T-cell count, **(B)** log-transformed NK-cell count, **(C)** low T-cell status, and **(D)** low NK-cell status. Panels show results for **(A)** log-transformed T-cell count, **(B)** log-transformed NK-cell count, **(C)** low T-cell status, and **(D)** low NK-cell status. Forest plot showing adjusted associations for key predictors, including disease category (obstruction/perforation-related acute abdomen vs. benign anorectal diseases), sex, age, NLR, and albumin, with log-transformed T-cell and NK-cell counts and with low T-cell and low NK-cell counts. Models were adjusted for disease category, sex, age, IL-6, CRP, albumin, and NLR. Low T-cell and low NK-cell counts were defined as the lowest quartile of the cohort and should be interpreted as exploratory outcomes. Results are from complete-case analyses (*n* = 623). Points represent adjusted geometric mean ratios (aGMRs) for log-linear models and odds ratios (ORs) for logistic models; horizontal lines indicate 95% confidence intervals. Low-count thresholds were ≤510 cells/μL for T cells and ≤89 cells/μL for NK cells.

In logistic regression analyses, low T-cell and low NK-cell counts were defined using cohort-specific lowest-quartile cutoffs (T cells 510.0 cells/μL; NK cells 89.0 cells/μL). This definition was exploratory and data-driven rather than based on external clinical reference intervals. Obstruction/perforation-related acute abdomen showed a borderline association with low T-cell count (OR = 2.939, 95% CI 1.001–8.627, *P* = 0.050), but not with low NK-cell count (OR = 1.026, 95% CI 0.424–2.482, *P* = 0.955). Given the borderline *P* value and wide confidence interval, the low T-cell association should be considered exploratory and its statistical significance differed across sensitivity analyses. NLR was independently associated with higher odds of both low T-cell count and low NK-cell count. Albumin was not significantly associated with low T-cell count (OR = 0.809, 95% CI 0.642–1.018, *P* = 0.070), although higher albumin was associated with lower odds of low NK-cell count (OR = 0.702, 95% CI 0.567–0.869, *P* = 0.001) (Table 2; Figure 3; Supplementary Table S5).

Multiple-imputation results are provided in Supplementary Table S6. Higher NLR remained associated with lower continuous T-cell and NK-cell counts and higher odds of cohort-defined low T-cell and low NK-cell status (T-cell count: aGMR = 0.717, 95% CI 0.672–0.766, *P* < 0.001; NK-cell count: aGMR = 0.749, 95% CI 0.690–0.815, *P* < 0.001; low T-cell status: OR = 3.529, 95% CI 2.464–5.054, *P* < 0.001; low NK-cell status: OR = 1.949, 95% CI 1.424–2.668, *P* < 0.001). Higher albumin was associated with higher NK-cell count (aGMR = 1.079, 95% CI 1.026–1.136, *P* = 0.003) and lower odds of both low T-cell status (OR = 0.756, 95% CI 0.617–0.926, *P* = 0.007) and low NK-cell status (OR = 0.703, 95% CI 0.583–0.848, *P* < 0.001), whereas its association with continuous T-cell count was less precise (aGMR = 1.036, 95% CI 0.996–1.078, *P* = 0.077). Unlike the complete-case continuous models, the imputed models identified associations of obstruction/perforation with lower T-cell count (aGMR = 0.822, 95% CI 0.706–0.958, *P* = 0.012) and lower NK-cell count (aGMR=0.821, 95% CI 0.677–0.995, *P* = 0.045). This category was also associated with low T-cell status (OR = 2.701, 95% CI 1.134–6.432, *P* = 0.025), but not low NK-cell status (OR = 1.567, 95% CI 0.720–3.413, *P* = 0.258). The continuous NK-cell estimate was close to the conventional significance threshold, and disease-category inferences were sensitive to the missing-data analysis.

In Firth penalized logistic sensitivity analyses, NLR remained associated with low T-cell and low NK-cell status (low T-cell status: OR = 2.967, 95% CI 2.011–4.376, *P* < 0.001; low NK-cell status: OR = 2.062, 95% CI 1.456–2.920, *P* < 0.001). The obstruction/perforation estimate for low T-cell status was imprecise and did not meet the conventional 0.05 significance threshold (OR = 2.733, 95% CI 0.959–7.788, *P* = 0.060) (Supplementary Table S6).

The diagnosis-text split identified 111 patients with obstruction/incarceration and 23 with perforation/peritonitis; 89 and 20 patients, respectively, were included in complete-case models. Neither subgroup was significantly associated with continuous T-cell or NK-cell counts after adjustment. Obstruction/incarceration was associated with cohort-defined low T-cell status (OR = 3.145, 95% CI 1.066–9.281, *P* = 0.038), whereas the corresponding estimate for perforation/peritonitis was imprecise (OR = 1.142, 95% CI 0.230–5.674, *P* = 0.871). These subgroup results were exploratory (Supplementary Table S6).

## Discussion

The principal finding of this study was that lower peripheral T-cell and NK-cell counts were more consistently associated with systemic inflammatory burden, particularly NLR, than with disease category after multivariable adjustment and sensitivity analyses. Albumin was associated with continuous lymphocyte counts and showed the most consistent relationship with NK-cell outcomes. The most prominent unadjusted immune-cell alterations were lower absolute T-cell and NK-cell counts in obstruction/perforation-related acute abdomen and acute infection/inflammation. However, disease-category associations were attenuated after adjustment for inflammatory burden, nutritional status, demographic structure, and related clinical factors. Disease-category inferences were sensitive to the missing-data approach: the imputed models identified associations between obstruction/perforation and continuous T-cell and NK-cell counts that did not meet the conventional significance threshold in the complete-case models. These differences argue against dismissing disease-category associations and support cautious interpretation.

These findings support the concept that lymphocyte subset testing can provide additional cellular immune information beyond routine inflammatory markers (11, 12). NLR is widely used as an integrated marker of neutrophil-dominant inflammation and relative lymphopenia (13–15). In our dataset, NLR showed the strongest and most consistent inverse relationship with both T-cell and NK-cell counts. Higher NLR was associated with lower circulating T-cell and NK-cell counts in this cohort, but these cross-sectional associations do not establish temporal direction or a causal mechanism. Because total lymphocyte count is the denominator of NLR, part of its inverse association with absolute lymphocyte subset counts may reflect mathematical coupling rather than an independent inflammatory effect.

The unadjusted association between obstruction/perforation-related acute abdomen and lower T-cell and NK-cell counts is clinically plausible. Obstruction and perforation can produce severe local tissue injury, bacterial translocation, peritoneal contamination, systemic inflammatory activation, and acute metabolic stress (16, 17). However, after adjustment for sex, age, inflammatory burden, and albumin, this disease-category association was attenuated and was not statistically significant in the complete-case linear T-cell or NK-cell models. These associations met the conventional significance threshold in the multiple-imputation models, although the NK-cell result was borderline and the diagnosis-text split yielded imprecise subgroup estimates. The differences between crude, complete-case, and imputed estimates indicate sensitivity to covariate adjustment and missing-data handling, but do not establish mediation by inflammatory or nutritional factors. Mechanical obstruction and gastrointestinal perforation may represent distinct inflammatory pathophysiological processes. In an exploratory sensitivity analysis separating obstruction/incarceration from perforation/peritonitis, the continuous T-cell and NK-cell models were broadly consistent with the original analysis, but subgroup estimates remained imprecise, particularly for perforation/peritonitis. Therefore, findings related to this category should be interpreted cautiously.

The relationship between albumin and lymphocyte subset counts is another important finding. Albumin is influenced by nutritional reserve, inflammatory suppression of hepatic synthesis, capillary leakage, and disease severity (8, 18). Higher albumin was independently associated with higher continuous T-cell and NK-cell counts and with lower odds of low NK-cell status; however, its association with low T-cell status did not reach statistical significance in the complete-case logistic model (19). Older age was associated with lower T-cell counts but higher NK-cell counts in the adjusted linear models. These findings may be interpreted in the context of age-related immune remodeling, including reduced T-cell production and reported increases in circulating NK-cell numbers (20, 21). However, higher NK-cell counts do not necessarily indicate preserved or enhanced immune function, and our data do not establish an immunosenescence-related mechanism. These findings support the broader concept of immunonutritional and age-related immune remodeling while also indicating that albumin should not be overinterpreted as a uniformly significant predictor across all categorical lymphocyte outcomes.

The gastrointestinal tumor group showed a different pattern. Although tumor patients were older, had longer hospital stays, and lower albumin and hemoglobin levels, their NK-cell counts were not the lowest among the categories. This may reflect heterogeneity in tumor stage, timing of testing, surgical indication, inflammatory status, and prior treatment. It also suggests that malignancy-related immune alterations in this cohort cannot be reduced to simple depletion of circulating NK cells (22, 23). Future analyses incorporating tumor stage, treatment history, and postoperative outcomes may clarify the clinical meaning of lymphocyte subset patterns in gastrointestinal cancer patients. Interpretation of the gastrointestinal tumor group is further limited by the absence of complete data on tumor stage, timing of blood sampling, operative approach, and neoadjuvant chemotherapy or radiotherapy, all of which may influence lymphocyte counts.

Our results have potential clinical implications. First, lymphocyte subset testing may help characterize cellular immune status in gastrointestinal surgical patients, particularly when interpreted alongside inflammatory burden and nutritional markers. Second, integrating T-cell and NK-cell counts with NLR and albumin may provide a practical immunoinflammatory profile that is easy to obtain from routine clinical data. Third, these markers may be useful for future risk-stratification research or for selecting patients who warrant closer perioperative monitoring, nutritional intervention, or infection surveillance. However, prospective studies with postoperative complication and survival outcomes are required before these markers can be used for decision-making.

### Strengths and limitations

The strengths of this study include the relatively large real-world gastrointestinal surgery cohort, integration of lymphocyte subset testing with inflammatory and nutritional markers, clinically interpretable disease classification, and multivariable modeling of both continuous lymphocyte counts and low-count status. The study also reflects routine hospital practice, which may improve clinical relevance.

Several limitations should be acknowledged. First, this was a single-center retrospective study, and causal relationships cannot be inferred. Second, disease categories were based on discharge diagnoses and may include clinical heterogeneity within each category. Third, low T-cell and low NK-cell counts were defined by cohort-specific lowest quartiles; these thresholds are exploratory and do not replace externally validated clinical reference intervals. Dichotomization discards information and may reduce statistical power. The cohort-derived thresholds require external validation against appropriate laboratory- and population-specific reference intervals before clinical interpretation. Fourth, PCT was measured in only 88 of 887 patients and was therefore summarized separately rather than retained as a continuous main-table variable (Supplementary Table S7). Fifth, the stoma-related postoperative subgroup was small (*n* = 16), and estimates for this category should be interpreted cautiously. Sixth, the complete-case multivariable analysis included 623 of 887 patients (70.2%); patients with and without complete covariate data differed in disease-category distribution and CRP availability/values (Supplementary Table S4), which could affect the generalizability of the regression findings. IL-6, CRP, and NLR reflect overlapping inflammatory processes. Although the maximum VIF of 4.141 did not suggest severe multicollinearity, individual coefficients may remain sensitive to model specification and should not be interpreted as isolated biological effects.

Clinical heterogeneity remains an important limitation. Disease categories were based on discharge diagnoses and do not fully characterize clinical presentation or operative complexity. Information on upper vs. lower gastrointestinal procedures, operative duration, surgical approach, stoma formation during the index admission, intervals from admission and surgery to blood sampling, corticosteroid exposure, transfusion, tumor stage, and neoadjuvant therapy could not be reliably retrieved for this cohort. These variables were therefore not included in adjusted or stratified analyses. The stoma-related discharge-diagnosis category should not be interpreted as identifying all patients who underwent stoma formation. Although the exploratory separation of obstruction/incarceration from perforation/peritonitis addressed one aspect of diagnostic heterogeneity, it did not resolve differences in operative characteristics or perioperative care. Center-specific management protocols may also influence the observed associations and limit generalizability. The additional statistical analyses do not eliminate residual confounding by these clinical factors.

The observed associations may reflect lymphocyte redistribution, impaired production, treatment-related effects, or residual confounding; these mechanisms were not directly assessed. Multiple imputation assumes missing at random conditional on the included variables and cannot exclude bias from missing-not-at-random mechanisms. Small-subgroup estimates also remain imprecise despite penalization.

## Conclusion

Continuous T-cell and NK-cell counts were the primary outcomes of this study and were associated with inflammatory and nutritional markers in a heterogeneous gastrointestinal surgery cohort. NLR associations were consistent across complete-case and sensitivity analyses, whereas albumin and disease-category estimates varied by outcome and analysis. Analyses of cohort-defined low-count status were secondary and exploratory and should not be interpreted as establishing clinical diagnostic thresholds. These observational findings do not establish causality. Prospective studies incorporating standardized blood-sampling times, detailed clinical presentations, anatomical site of surgery, operative duration, stoma status, and perioperative management are needed to assess clinical applicability.

## Data Availability

The raw data supporting the conclusions of this article will be made available by the authors, without undue reservation.

## Author's note

This study was reported in accordance with the STROBE guideline for observational studies. A completed STROBE checklist is provided as Supplementary Material.

## Supplementary material

SUPPLEMENTARY FIGURE S1 Flow cytometry gating strategy for peripheral blood lymphocyte subset analysis.
