# Supplementary material for: Lower peripheral T- and NK-cell counts are associated with systemic inflammation and nutritional status in hospitalized gastrointestinal surgery patients
Source: Front Med (Lausanne). 2026 Oct 21;13:1916752. doi: 10.3389/fmed.2026.1916752 (PMC13730929; doi:10.3389/fmed.2026.1916752)
Supplement: Supplementary file 1 [file Image1.pdf]

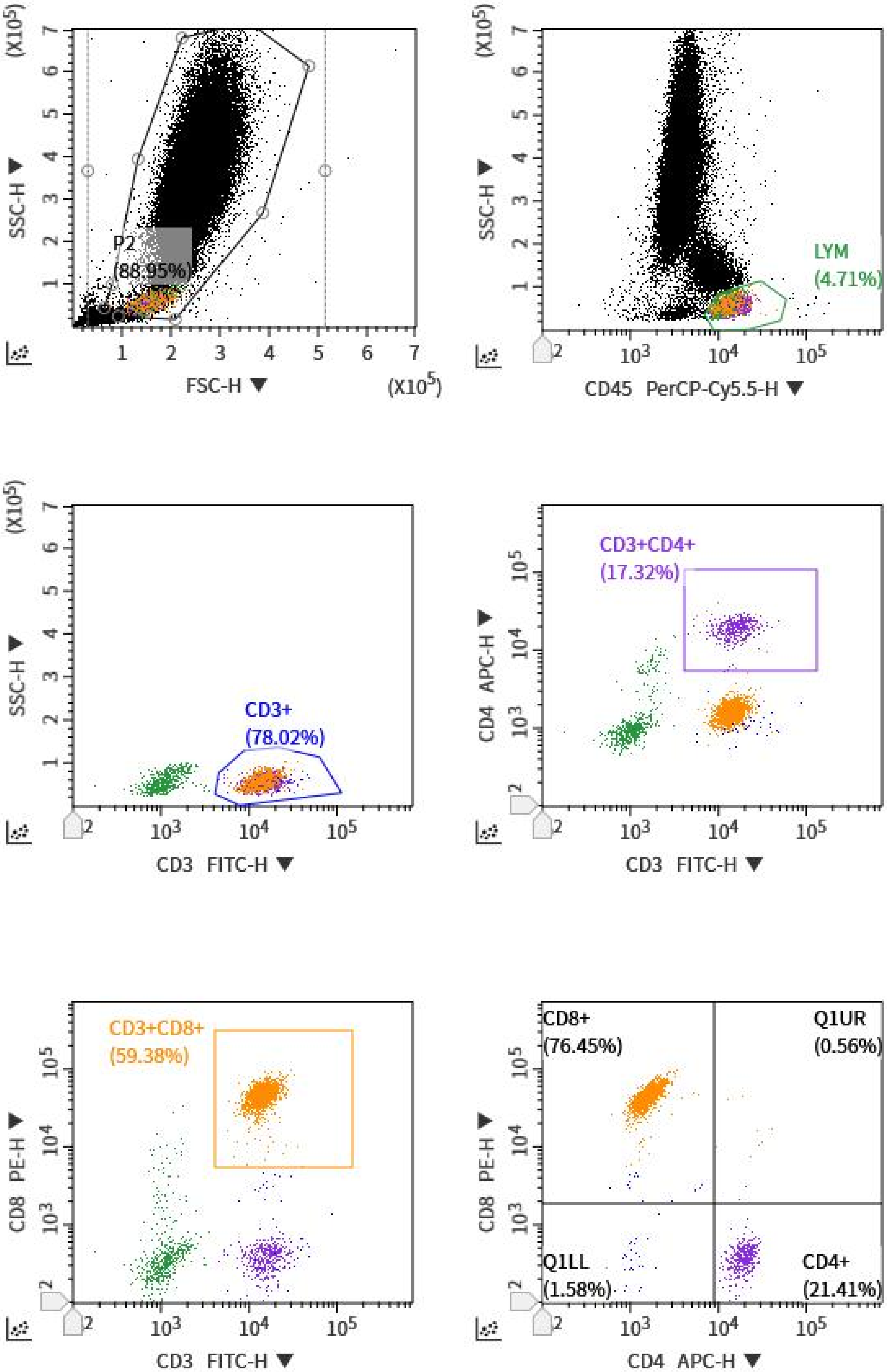
P2
(88.95%)
SSC-H
FSC-H
(X10^5)
LYM
(4.71%)
CD45 PerCP-Cy5.5-H
CD3+
(78.02%)
CD3 FITC-H
CD3+CD4+
(17.32%)
CD4 APC-H
CD3+CD8+
(59.38%)
CD8 PE-H
CD8+
(76.45%)
Q1UR
(0.56%)
Q1LL
(1.58%)
CD4+
(21.41%)

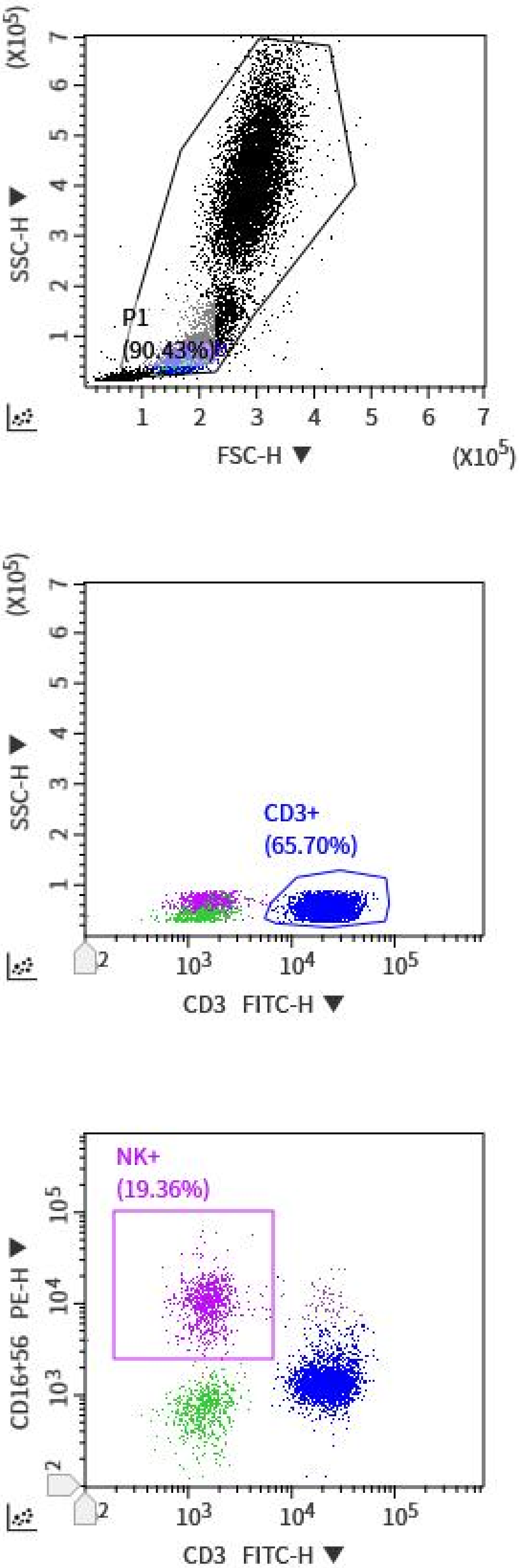
P1
(90.43%)
SSC-H
FSC-H
(X10^5)
LYM
(30.69%)
CD45 PerCP-Cy5.5-H
CD3+
(65.70%)
CD3 FITC-H
B+
(14.91%)
CD19 APC-H
NK+
(19.36%)
CD16+56 PE-H
NKT
(1.91%)
