# Supplementary material for: Lower peripheral T- and NK-cell counts are associated with systemic inflammation and nutritional status in hospitalized gastrointestinal surgery patients
Source: Front Med (Lausanne). 2026 Oct 21;13:1916752. doi: 10.3389/fmed.2026.1916752 (PMC13730929; doi:10.3389/fmed.2026.1916752)
Supplement: Supplementary file 2 [file Supplementaryfile1.docx]

**STROBE Statement Checklist**

Manuscript: Lower peripheral T- and NK-cell counts are associated with systemic inflammation and nutritional status in hospitalized gastrointestinal surgery patients

*This checklist was completed for a retrospective observational study using routinely collected hospital data. Locations are provided by manuscript section because page numbers may change during journal formatting.*

| **Section** | **Item** | **STROBE recommendation** | **Reported location** | **Comment** |
| --- | --- | --- | --- | --- |
| Title and abstract | 1a | Indicate the study's design with a commonly used term in the title or the abstract. | Abstract, Methods | The abstract identifies the study as a retrospective observational study of hospitalized gastrointestinal surgery patients. |
| Title and abstract | 1b | Provide in the abstract an informative and balanced summary of what was done and what was found. | Abstract, Methods; Results; Conclusion | The abstract reports the 887-patient cohort, 623 complete cases, and pooled T-cell and NK-cell estimates from 50 imputed datasets. It distinguishes primary continuous outcomes from secondary exploratory low-count outcomes and notes sensitivity to missing-data handling. |
| Introduction | 2 | Explain the scientific background and rationale for the investigation being reported. | Introduction, paragraphs 1-3 | The introduction describes inflammation, lymphocyte subsets, nutritional markers, and their relevance in gastrointestinal surgical patients. |
| Introduction | 3 | State specific objectives, including any prespecified hypotheses. | Introduction, final paragraph | The study objective and hypothesis are explicitly stated. |
| Methods | 4 | Present key elements of study design early in the paper. | Methods, Study Design and Patients | The study is described as a single-center retrospective observational study. |
| Methods | 5 | Describe the setting, locations, and relevant dates, including periods of recruitment, exposure, follow-up, and data collection. | Methods, Study Design and Patients | The setting is the Department of Gastrointestinal Surgery at the First Hospital of Changsha; admission dates were January 2024 to May 2025. |
| Methods | 6a | Give the eligibility criteria, and the sources and methods of selection of participants. | Methods, Study Design and Patients | Inclusion and exclusion criteria are listed; patients were selected from hospitalized patients with lymphocyte subset testing and discharge diagnoses. |
| Methods | 6b | For matched studies, give matching criteria and number of exposed and unexposed. | Not applicable | This was not a matched study. |
| Methods | 7 | Clearly define all outcomes, exposures, predictors, potential confounders, and effect modifiers. Give diagnostic criteria, if applicable. | Methods, Disease Classification; Laboratory Variables; Statistical Analysis | Continuous T-cell and NK-cell counts are primary outcomes. Cohort-defined low-count outcomes are secondary and exploratory. Disease categories, measured covariates, and outcome definitions are described; unavailable clinical factors are identified in the Limitations. |
| Methods | 8 | For each variable of interest, give sources of data and details of methods of assessment. Describe comparability of assessment methods if there is more than one group. | Methods, Laboratory Variables; Flow Cytometric TBNK Assay; Supplementary Figure S1 | Routine laboratory variables and flow cytometry procedures are described. Methods cites the supplementary gating figure. |
| Methods | 9 | Describe any efforts to address potential sources of bias. | Methods, Study Design and Patients; Statistical Analysis; Results, Multivariable Analysis; Strengths and Limitations | Duplicate handling, date-based matching, covariate adjustment, complete/incomplete-case comparison, multiple imputation, Firth regression, and an exploratory diagnosis split are reported. Residual clinical confounding and missing-not-at-random bias remain possible. |
| Methods | 10 | Explain how the study size was arrived at. | Methods, Study Design and Patients; Results, Study Population and Disease Categories | Consecutive eligible tested inpatients were considered during the study period. The cleaned dataset contained 888 records; one missing diagnosis was excluded, leaving 887 observations. No formal sample-size calculation is reported. |
| Methods | 11 | Explain how quantitative variables were handled in the analyses. If applicable, describe which groupings were chosen and why. | Methods, Statistical Analysis; Results, Multivariable Analysis | Continuous variables are summarized as medians and IQRs. Counts are log-transformed; IL-6, CRP, and NLR use log1p transformations. Age is modeled per 10 years and albumin per 5 g/L. Low-count thresholds are cohort-specific and exploratory. |
| Methods | 12a | Describe all statistical methods, including those used to control for confounding. | Methods, Statistical Analysis; Results, Multivariable Analysis | Group comparisons, correlations, linear and logistic regression, covariate adjustment, Bonferroni and FDR corrections, multiple imputation, and Firth regression are described. FDR and VIF details also appear in Results. |
| Methods | 12b | Describe any methods used to examine subgroups and interactions. | Methods, Statistical Analysis; Results, Multivariable Analysis; Supplementary Table S6 | An exploratory diagnosis-keyword split distinguishes obstruction/incarceration from perforation/peritonitis, with perforation-related keywords taking precedence. No formal interaction analyses are reported. |
| Methods | 12c | Explain how missing data were addressed. | Methods, Statistical Analysis; Results, Multivariable Analysis; Supplementary Tables S4 and S6 | Complete-case models include 623 patients; 264 have incomplete covariates. MICE imputes four covariates using parameter draws and predictive mean matching, retaining 50 datasets. Estimates are pooled using Rubin rules and Barnard-Rubin degrees of freedom. Donor-value checks and an independent-seed run are described. |
| Methods | 12d | If applicable, explain how loss to follow-up was addressed. | Not applicable | The study used routinely collected inpatient laboratory data and did not include longitudinal follow-up. |
| Methods | 12e | Describe any sensitivity analyses. | Methods, Statistical Analysis; Results, Multivariable Analysis; Supplementary Table S6 | Sensitivity analyses comprise multiple imputation, complete-case Firth logistic regression with expected-information Wald inference, and the obstruction/perforation diagnosis split. Their exploratory nature is stated. |
| Results | 13a | Report numbers of individuals at each stage of study, for example potentially eligible, examined for eligibility, confirmed eligible, included in the study, completing follow-up, and analyzed. | Results, Study Population and Disease Categories; Multivariable Analysis | The cleaned dataset contains 888 records; one missing diagnosis is excluded. Analyses include 887 observations overall and 623 complete cases; 264 have incomplete covariates. Multiple imputation retains the full cohort. |
| Results | 13b | Give reasons for non-participation at each stage. | Results, Study Population and Disease Categories | The only exclusion from the cleaned dataset was missing diagnosis. |
| Results | 13c | Consider use of a flow diagram. | Not provided | A flow diagram was not included; participant flow is described in text. |
| Results | 14a | Give characteristics of study participants and information on exposures and potential confounders. | Results, Study Population; Table 1; Supplementary Table S1 | Baseline characteristics, disease categories, sex, age, LOS, and laboratory variables are summarized. |
| Results | 14b | Indicate number of participants with missing data for each variable of interest. | Table 1 notes; Supplementary Tables S1b, S4 and S6 | Available sample sizes are reported by variable and disease category. The complete/incomplete-case comparison and sensitivity workbook provide additional missing-data information. |
| Results | 14c | Summarise follow-up time, if applicable. | Not applicable | No follow-up-time analysis was performed; LOS was reported as a clinical descriptor. |
| Results | 15 | Report numbers of outcome events or summary measures. | Results, Disease Categories Showed Distinct Lymphocyte Subset Profiles; Multivariable Analysis; Supplementary Tables S1, S5b and S6 | Count distributions and low-count definitions are reported. Supplementary Table S5b reports 160 low T-cell and 167 low NK-cell events among the 623 complete cases; sensitivity results are in S6. |
| Results | 16a | Give unadjusted estimates and, if applicable, confounder-adjusted estimates and their precision. Make clear which confounders were adjusted for and why they were included. | Results; Table 2; Supplementary Tables S1, S2, S5 and S6 | Unadjusted group summaries and pairwise comparisons accompany adjusted aGMRs and ORs with 95% CIs and P values. Model covariates are specified. Complete-case and imputed estimates are distinguished. |
| Results | 16b | Report category boundaries when continuous variables were categorized. | Methods, Statistical Analysis; Results, Multivariable Analysis; Figure 3 legend; Supplementary Table S5b | Low T-cell and NK-cell status is defined as counts at or below 510 and 89 cells/μL, respectively. These cohort-derived thresholds are secondary and exploratory, not clinical diagnostic limits. |
| Results | 16c | If relevant, consider translating estimates of relative risk into absolute risk for a meaningful time period. | Not applicable | The study did not estimate time-dependent risks or prospective incidence rates. |
| Results | 17 | Report other analyses done, such as analyses of subgroups and interactions, and sensitivity analyses. | Results; Supplementary Tables S2–S7 | Additional analyses include pairwise comparisons (S2), correlations (S3), complete/incomplete-case comparison (S4), full regression models (S5), imputation/Firth/diagnosis-split sensitivity analyses (S6), and PCT availability summaries (S7). |
| Discussion | 18 | Summarise key results with reference to study objectives. | Discussion, opening paragraph; Conclusion | Continuous counts are the primary outcomes. NLR associations are consistent across analyses; albumin and disease-category estimates vary by outcome and analysis. Low-count outcomes are secondary and exploratory. |
| Discussion | 19 | Discuss limitations, taking into account sources of potential bias or imprecision. | Strengths and Limitations | Limitations include retrospective single-center design, clinical heterogeneity, unavailable operative/treatment data, exploratory thresholds, sparse subgroups, missing data, possible MNAR bias, overlapping inflammatory markers, and residual confounding. Penalization does not eliminate imprecision. |
| Discussion | 20 | Give a cautious overall interpretation of results considering objectives, limitations, multiplicity, and evidence from similar studies. | Discussion; Strengths and Limitations; Conclusion | Interpretation distinguishes complete-case from imputed estimates and acknowledges missing-data sensitivity. Findings do not establish causality or validated clinical thresholds; clinical application requires prospective validation. |
| Discussion | 21 | Discuss the generalisability of the study results. | Strengths and Limitations; Conclusion | Generalizability is limited by the single-center tested-inpatient cohort, missing covariates, clinical heterogeneity, and center-specific management. Prospective validation should incorporate sampling timing, operative characteristics, stoma status, and perioperative care. |
| Other information | 22 | Give the source of funding and the role of the funders for the present study and, if applicable, for the original study on which the present article is based. | Funding | Funding sources and grant numbers are listed. The role of the funders is not explicitly stated in the current manuscript. |
