# Supplementary material for: Lower peripheral T- and NK-cell counts are associated with systemic inflammation and nutritional status in hospitalized gastrointestinal surgery patients
Source: Front Med (Lausanne). 2026 Oct 21;13:1916752. doi: 10.3389/fmed.2026.1916752 (PMC13730929; doi:10.3389/fmed.2026.1916752)
Supplement: Supplementary file 3 [file Table1.docx]

**Supplementary Tables**

Supplementary Table S1a. Full group comparison of lymphocyte subset, inflammatory, and nutritional markers.

| Variable | P | INF (n=258) | TUM (n=150) | OPA (n=134) | ANO (n=74) | HER (n=32) | POL (n=136) | STO (n=16) | OTH (n=87) |
| --- | --- | --- | --- | --- | --- | --- | --- | --- | --- |
| T cells, % | <0.001 | 68.85 (62.53-75.82) | 69.03 (64.52-75.75) | 64.78 (54.78-73.37) | 71.40 (65.75-75.69) | 69.92 (61.07-74.47) | 71.16 (66.22-76.92) | 71.84 (68.10-77.25) | 72.91 (66.59-78.03) |
| T cells, cells/μL | <0.001 | 651.5 (476.5-951.0) | 801.5 (568.5-995.5) | 492.0 (308.8-764.3) | 853.5 (646.8-1184.3) | 748.0 (496.0-1007.3) | 935.5 (685.0-1146.0) | 943.0 (664.0-1096.3) | 877.0 (670.5-1175.5) |
| NK cells, % | <0.001 | 12.88 (8.41-19.35) | 15.92 (11.83-22.01) | 14.45 (10.28-22.78) | 13.16 (9.09-18.52) | 15.71 (11.43-21.72) | 12.21 (7.84-18.72) | 13.07 (8.96-14.16) | 11.63 (7.89-16.16) |
| NK cells, cells/μL | <0.001 | 124.0 (80.50-177.8) | 175.0 (117.3-274.3) | 110.0 (70.25-175.0) | 168.5 (122.0-234.5) | 138.0 (101.3-215.0) | 142.5 (103.0-245.3) | 154.0 (94.50-197.5) | 133.0 (87.00-205.0) |
| B cells, % | <0.001 | 14.45 (10.89-18.07) | 11.05 (7.34-14.52) | 14.30 (10.03-20.51) | 12.80 (11.06-16.47) | 12.43 (10.07-16.23) | 12.27 (9.07-16.71) | 11.43 (7.08-14.48) | 12.79 (9.13-17.10) |
| B cells, cells/μL | <0.001 | 140.0 (93.00-198.8) | 117.0 (74.50-173.0) | 107.5 (76.00-174.5) | 164.5 (118.8-211.8) | 136.0 (76.25-198.5) | 153.0 (99.50-245.8) | 138.0 (73.75-176.5) | 142.8 (88.00-235.0) |
| NKT cells, % | 0.172 | 3.22 (2.11-4.36) | 2.79 (1.89-3.66) | 2.64 (1.80-3.99) | 2.88 (2.03-4.06) | 2.38 (1.89-3.63) | 2.98 (1.71-3.83) | 2.58 (1.47-3.77) | 2.99 (2.16-3.67) |
| NKT cells, cells/μL | <0.001 | 29.50 (17.00-47.75) | 30.00 (18.00-46.50) | 21.00 (12.00-34.00) | 34.00 (23.50-51.75) | 30.50 (15.50-37.25) | 32.00 (18.00-50.00) | 21.50 (17.75-52.50) | 32.00 (19.00-49.00) |
| IL-6, pg/mL | <0.001 | 29.30 (13.40-76.40) | 9.35 (4.20-24.40) | 20.10 (7.70-51.10) | 3.90 (2.65-5.80) | 4.50 (3.30-7.20) | 10.10 (4.40-37.55) | 4.80 (4.03-5.60) | 9.20 (4.60-22.80) |
| CRP, mg/L | <0.001 | 23.15 (6.20-51.45) | 4.73 (1.65-18.66) | 6.80 (2.52-33.27) | 1.10 (0.45-2.12) | 1.66 (1.06-5.05) | 4.94 (1.27-34.03) | 1.41 (0.91-4.87) | 3.63 (1.15-23.93) |
| WBC count | <0.001 | 9.24 (7.00-11.82) | 6.74 (5.24-8.48) | 6.80 (4.92-9.70) | 5.90 (5.12-7.49) | 5.99 (4.73-7.80) | 6.42 (5.36-7.96) | 5.81 (5.64-6.06) | 6.63 (5.36-8.20) |
| Neutrophil count | <0.001 | 7.14 (4.94-9.67) | 4.70 (3.31-5.96) | 5.31 (3.04-7.50) | 3.65 (3.10-4.81) | 3.81 (2.74-4.93) | 4.14 (3.10-5.93) | 3.73 (3.00-4.41) | 4.55 (3.24-5.76) |
| Lymphocyte count | <0.001 | 1.22 (0.89-1.69) | 1.33 (0.97-1.62) | 0.95 (0.67-1.42) | 1.56 (1.18-1.99) | 1.37 (1.00-1.79) | 1.50 (1.15-1.86) | 1.56 (0.98-1.77) | 1.37 (1.09-1.83) |
| NLR | <0.001 | 5.68 (3.37-9.56) | 3.33 (2.45-5.48) | 4.95 (2.50-9.72) | 2.52 (1.83-3.30) | 2.48 (1.89-5.13) | 2.74 (1.88-4.74) | 2.31 (1.82-4.05) | 3.04 (2.12-4.88) |
| LMR | <0.001 | 1.90 (1.35-2.86) | 3.02 (1.90-3.73) | 2.05 (1.24-3.48) | 3.59 (2.91-4.49) | 2.59 (2.02-3.68) | 3.28 (2.09-4.24) | 3.21 (2.63-4.09) | 2.59 (1.88-3.94) |
| PLR | <0.001 | 180.3 (127.1-250.0) | 188.7 (135.7-263.0) | 210.5 (158.5-335.5) | 147.2 (111.4-186.5) | 129.0 (113.8-211.5) | 154.5 (114.6-198.4) | 136.6 (105.2-241.5) | 164.2 (127.7-203.3) |
| Albumin, g/L | <0.001 | 43.10 (39.20-45.45) | 41.05 (37.10-43.02) | 40.50 (36.00-44.60) | 44.25 (41.50-46.95) | 41.60 (38.65-44.00) | 42.20 (40.10-44.30) | 42.80 (39.42-44.60) | 41.90 (38.20-44.70) |
| Total protein | <0.001 | 68.20 (63.75-72.55) | 65.75 (61.35-69.55) | 65.40 (58.30-70.30) | 70.15 (65.20-73.72) | 68.80 (64.40-72.05) | 67.30 (64.10-71.60) | 68.00 (65.60-72.30) | 68.20 (62.70-72.10) |
| Hemoglobin, g/L | <0.001 | 127.0 (116.0-140.0) | 125.0 (103.0-139.0) | 123.0 (112.0-133.0) | 137.0 (127.0-147.0) | 137.0 (124.0-143.0) | 133.0 (122.0-145.0) | 127.0 (112.0-137.8) | 132.0 (111.0-143.0) |
| RBC count | 0.003 | 4.29 (3.90-4.72) | 4.22 (3.69-4.78) | 4.14 (3.72-4.59) | 4.60 (4.22-4.89) | 4.48 (3.97-4.75) | 4.33 (3.94-4.72) | 4.29 (3.95-4.75) | 4.38 (3.78-4.88) |
| Platelet count | 0.018 | 224.0 (181.0-263.0) | 237.0 (198.0-308.0) | 210.0 (166.5-260.5) | 235.0 (203.0-279.0) | 221.0 (189.5-249.0) | 234.0 (188.0-277.0) | 228.0 (199.3-247.8) | 232.0 (180.0-289.0) |
| Total cholesterol | 0.098 | 4.23 (3.68-4.76) | 4.08 (3.44-4.80) | 4.16 (3.41-4.94) | 4.48 (3.98-5.23) | 4.75 (3.66-5.00) | 4.41 (3.92-4.98) | 4.54 (3.93-5.21) | 4.06 (3.22-5.01) |
| Triglycerides | <0.001 | 0.99 (0.70-1.60) | 1.20 (1.01-1.75) | 1.15 (0.84-1.47) | 1.33 (0.92-2.06) | 1.92 (0.87-3.02) | 1.27 (0.94-1.79) | 1.36 (0.97-1.67) | 1.21 (0.94-1.81) |

Note. Values are median (interquartile range) unless otherwise indicated. Group sizes are shown in column headers. Available sample sizes varied across laboratory variables because of missing or unreported tests; detailed available n values are provided in Supplementary Table S1b. INF, acute infection/inflammation; TUM, GI tumors; OPA, obstruction/perforation acute abdomen; ANO, benign anorectal diseases; HER, hernia/abdominal wall diseases; POL, benign polyps/diverticular disease; STO, stoma-related conditions; OTH, other diseases.

Supplementary Table S1b. Available sample size matrix for Supplementary Table S1a.

| Variable | INF | TUM | OPA | ANO | HER | POL | STO | OTH |
| --- | --- | --- | --- | --- | --- | --- | --- | --- |
| T cells, % | 258 | 150 | 134 | 74 | 32 | 136 | 16 | 87 |
| T cells, cells/μL | 258 | 150 | 134 | 74 | 32 | 136 | 16 | 87 |
| NK cells, % | 258 | 150 | 134 | 74 | 32 | 135 | 16 | 87 |
| NK cells, cells/μL | 258 | 150 | 134 | 74 | 32 | 136 | 16 | 87 |
| B cells, % | 258 | 150 | 134 | 74 | 32 | 136 | 16 | 87 |
| B cells, cells/μL | 258 | 150 | 134 | 74 | 32 | 136 | 16 | 87 |
| NKT cells, % | 258 | 150 | 134 | 74 | 32 | 136 | 16 | 87 |
| NKT cells, cells/μL | 258 | 150 | 134 | 74 | 32 | 136 | 16 | 87 |
| IL-6, pg/mL | 213 | 130 | 129 | 55 | 25 | 123 | 14 | 75 |
| CRP, mg/L | 185 | 117 | 115 | 58 | 27 | 110 | 15 | 72 |
| WBC count | 226 | 141 | 131 | 69 | 31 | 125 | 16 | 85 |
| Neutrophil count | 226 | 141 | 131 | 69 | 31 | 125 | 16 | 85 |
| Lymphocyte count | 226 | 141 | 131 | 69 | 31 | 125 | 16 | 85 |
| NLR | 226 | 141 | 131 | 69 | 31 | 125 | 16 | 85 |
| LMR | 226 | 141 | 130 | 69 | 31 | 125 | 16 | 85 |
| PLR | 225 | 141 | 131 | 69 | 31 | 125 | 16 | 85 |
| Albumin, g/L | 227 | 144 | 133 | 70 | 31 | 129 | 16 | 85 |
| Total protein | 227 | 144 | 133 | 70 | 31 | 129 | 16 | 85 |
| Hemoglobin, g/L | 226 | 141 | 131 | 69 | 31 | 125 | 16 | 85 |
| RBC count | 226 | 141 | 131 | 69 | 31 | 125 | 16 | 85 |
| Platelet count | 225 | 141 | 131 | 69 | 31 | 125 | 16 | 85 |
| Total cholesterol | 158 | 133 | 106 | 47 | 23 | 109 | 14 | 68 |
| Triglycerides | 157 | 133 | 106 | 46 | 23 | 109 | 14 | 68 |

Note. Values indicate the number of available observations for each variable and disease category. Blank or unreported laboratory values were treated as missing.

Supplementary Table S2. Pairwise comparisons versus benign anorectal diseases after Bonferroni correction

| Variable | Comparison group | Benign anorectal diseases | Comparison group value | Raw P | Bonferroni-adjusted P |
| --- | --- | --- | --- | --- | --- |
| T cells, cells/μL | Acute infection / inflammation | 853.5 (646.8-1184.3); n=74 | 651.5 (476.5-951.0); n=258 | <0.001 | <0.001 |
|  | GI tumors | 853.5 (646.8-1184.3); n=74 | 801.5 (568.5-995.5); n=150 | 0.120 | 0.837 |
|  | Obstruction / perforation acute abdomen | 853.5 (646.8-1184.3); n=74 | 492.0 (308.8-764.3); n=134 | <0.001 | <0.001 |
|  | Hernia / abdominal wall diseases | 853.5 (646.8-1184.3); n=74 | 748.0 (496.0-1007.3); n=32 | 0.049 | 0.341 |
|  | Benign polyps / diverticular disease | 853.5 (646.8-1184.3); n=74 | 935.5 (685.0-1146.0); n=136 | 0.323 | 1.000 |
|  | Stoma-related conditions | 853.5 (646.8-1184.3); n=74 | 943.0 (664.0-1096.3); n=16 | 0.966 | 1.000 |
|  | Other diseases | 853.5 (646.8-1184.3); n=74 | 877.0 (670.5-1175.5); n=87 | 0.831 | 1.000 |
| NK cells, cells/μL | Acute infection / inflammation | 168.5 (122.0-234.5); n=74 | 124.0 (80.50-177.8); n=258 | <0.001 | 0.003 |
|  | GI tumors | 168.5 (122.0-234.5); n=74 | 175.0 (117.3-274.3); n=150 | 0.450 | 1.000 |
|  | Obstruction / perforation acute abdomen | 168.5 (122.0-234.5); n=74 | 110.0 (70.25-175.0); n=134 | <0.001 | 0.001 |
|  | Hernia / abdominal wall diseases | 168.5 (122.0-234.5); n=74 | 138.0 (101.3-215.0); n=32 | 0.332 | 1.000 |
|  | Benign polyps / diverticular disease | 168.5 (122.0-234.5); n=74 | 142.5 (103.0-245.3); n=136 | 0.389 | 1.000 |
|  | Stoma-related conditions | 168.5 (122.0-234.5); n=74 | 154.0 (94.50-197.5); n=16 | 0.378 | 1.000 |
|  | Other diseases | 168.5 (122.0-234.5); n=74 | 133.0 (87.00-205.0); n=87 | 0.026 | 0.179 |
| IL-6, pg/mL | Acute infection / inflammation | 3.90 (2.65-5.80); n=55 | 29.30 (13.40-76.40); n=213 | <0.001 | <0.001 |
|  | GI tumors | 3.90 (2.65-5.80); n=55 | 9.35 (4.20-24.40); n=130 | <0.001 | <0.001 |
|  | Obstruction / perforation acute abdomen | 3.90 (2.65-5.80); n=55 | 20.10 (7.70-51.10); n=129 | <0.001 | <0.001 |
|  | Hernia / abdominal wall diseases | 3.90 (2.65-5.80); n=55 | 4.50 (3.30-7.20); n=25 | 0.103 | 0.722 |
|  | Benign polyps / diverticular disease | 3.90 (2.65-5.80); n=55 | 10.10 (4.40-37.55); n=123 | <0.001 | <0.001 |
|  | Stoma-related conditions | 3.90 (2.65-5.80); n=55 | 4.80 (4.03-5.60); n=14 | 0.140 | 0.977 |
|  | Other diseases | 3.90 (2.65-5.80); n=55 | 9.20 (4.60-22.80); n=75 | <0.001 | <0.001 |
| CRP, mg/L | Acute infection / inflammation | 1.10 (0.45-2.12); n=58 | 23.15 (6.20-51.45); n=185 | <0.001 | <0.001 |
|  | GI tumors | 1.10 (0.45-2.12); n=58 | 4.73 (1.65-18.66); n=117 | <0.001 | <0.001 |
|  | Obstruction / perforation acute abdomen | 1.10 (0.45-2.12); n=58 | 6.80 (2.52-33.27); n=115 | <0.001 | <0.001 |
|  | Hernia / abdominal wall diseases | 1.10 (0.45-2.12); n=58 | 1.66 (1.06-5.05); n=27 | 0.008 | 0.054 |
|  | Benign polyps / diverticular disease | 1.10 (0.45-2.12); n=58 | 4.94 (1.27-34.03); n=110 | <0.001 | <0.001 |
|  | Stoma-related conditions | 1.10 (0.45-2.12); n=58 | 1.41 (0.91-4.87); n=15 | 0.299 | 1.000 |
|  | Other diseases | 1.10 (0.45-2.12); n=58 | 3.63 (1.15-23.93); n=72 | <0.001 | <0.001 |
| NLR | Acute infection / inflammation | 2.52 (1.83-3.30); n=69 | 5.68 (3.37-9.56); n=226 | <0.001 | <0.001 |
|  | GI tumors | 2.52 (1.83-3.30); n=69 | 3.33 (2.45-5.48); n=141 | <0.001 | <0.001 |
|  | Obstruction / perforation acute abdomen | 2.52 (1.83-3.30); n=69 | 4.95 (2.50-9.72); n=131 | <0.001 | <0.001 |
|  | Hernia / abdominal wall diseases | 2.52 (1.83-3.30); n=69 | 2.48 (1.89-5.13); n=31 | 0.404 | 1.000 |
|  | Benign polyps / diverticular disease | 2.52 (1.83-3.30); n=69 | 2.74 (1.88-4.74); n=125 | 0.250 | 1.000 |
|  | Stoma-related conditions | 2.52 (1.83-3.30); n=69 | 2.31 (1.82-4.05); n=16 | 0.991 | 1.000 |
|  | Other diseases | 2.52 (1.83-3.30); n=69 | 3.04 (2.12-4.88); n=85 | 0.018 | 0.127 |
| LMR | Acute infection / inflammation | 3.59 (2.91-4.49); n=69 | 1.90 (1.35-2.86); n=226 | <0.001 | <0.001 |
|  | GI tumors | 3.59 (2.91-4.49); n=69 | 3.02 (1.90-3.73); n=141 | <0.001 | 0.002 |
|  | Obstruction / perforation acute abdomen | 3.59 (2.91-4.49); n=69 | 2.05 (1.24-3.48); n=130 | <0.001 | <0.001 |
|  | Hernia / abdominal wall diseases | 3.59 (2.91-4.49); n=69 | 2.59 (2.02-3.68); n=31 | 0.007 | 0.051 |
|  | Benign polyps / diverticular disease | 3.59 (2.91-4.49); n=69 | 3.28 (2.09-4.24); n=125 | 0.024 | 0.167 |
|  | Stoma-related conditions | 3.59 (2.91-4.49); n=69 | 3.21 (2.63-4.09); n=16 | 0.342 | 1.000 |
|  | Other diseases | 3.59 (2.91-4.49); n=69 | 2.59 (1.88-3.94); n=85 | 0.002 | 0.014 |
| Albumin, g/L | Acute infection / inflammation | 44.25 (41.50-46.95); n=70 | 43.10 (39.20-45.45); n=227 | 0.005 | 0.038 |
|  | GI tumors | 44.25 (41.50-46.95); n=70 | 41.05 (37.10-43.02); n=144 | <0.001 | <0.001 |
|  | Obstruction / perforation acute abdomen | 44.25 (41.50-46.95); n=70 | 40.50 (36.00-44.60); n=133 | <0.001 | <0.001 |
|  | Hernia / abdominal wall diseases | 44.25 (41.50-46.95); n=70 | 41.60 (38.65-44.00); n=31 | 0.001 | 0.009 |
|  | Benign polyps / diverticular disease | 44.25 (41.50-46.95); n=70 | 42.20 (40.10-44.30); n=129 | <0.001 | <0.001 |
|  | Stoma-related conditions | 44.25 (41.50-46.95); n=70 | 42.80 (39.42-44.60); n=16 | 0.030 | 0.213 |
|  | Other diseases | 44.25 (41.50-46.95); n=70 | 41.90 (38.20-44.70); n=85 | <0.001 | 0.003 |

Note. Values are median (IQR); n indicates available observations. Benign anorectal diseases were used as the reference group for all pairwise comparisons. Raw P values were derived from pairwise Wilcoxon rank-sum tests, and Bonferroni-adjusted P values were calculated across the seven comparisons within each variable.

Supplementary Table S3. Spearman correlations between lymphocyte subset counts and clinical markers.

| X | Y | n | Spearman r | P | FDR-adjusted P |
| --- | --- | --- | --- | --- | --- |
| T cells, cells/μL | IL-6, pg/mL | 764 | -0.336 | <0.001 | <0.001 |
|  | CRP, mg/L | 699 | -0.233 | <0.001 | <0.001 |
|  | WBC count | 824 | 0.038 | 0.274 | 0.302 |
|  | NLR | 824 | -0.487 | <0.001 | <0.001 |
|  | LMR | 823 | 0.487 | <0.001 | <0.001 |
|  | Albumin, g/L | 835 | 0.200 | <0.001 | <0.001 |
|  | Hemoglobin, g/L | 824 | 0.201 | <0.001 | <0.001 |
|  | Length of stay, days | 844 | -0.095 | 0.006 | 0.007 |
| NK cells, cells/μL | IL-6, pg/mL | 764 | -0.186 | <0.001 | <0.001 |
|  | CRP, mg/L | 699 | -0.199 | <0.001 | <0.001 |
|  | WBC count | 824 | -0.026 | 0.463 | 0.476 |
|  | NLR | 824 | -0.262 | <0.001 | <0.001 |
|  | LMR | 823 | 0.246 | <0.001 | <0.001 |
|  | Albumin, g/L | 835 | 0.074 | 0.033 | 0.040 |
|  | Hemoglobin, g/L | 824 | 0.131 | <0.001 | <0.001 |
|  | Length of stay, days | 844 | 0.053 | 0.123 | 0.141 |
| B cells, cells/μL | IL-6, pg/mL | 764 | -0.173 | <0.001 | <0.001 |
|  | CRP, mg/L | 699 | -0.128 | <0.001 | <0.001 |
|  | WBC count | 824 | 0.135 | <0.001 | <0.001 |
|  | NLR | 824 | -0.306 | <0.001 | <0.001 |
|  | LMR | 823 | 0.304 | <0.001 | <0.001 |
|  | Albumin, g/L | 835 | 0.208 | <0.001 | <0.001 |
|  | Hemoglobin, g/L | 824 | 0.216 | <0.001 | <0.001 |
|  | Length of stay, days | 844 | -0.153 | <0.001 | <0.001 |
| NKT cells, cells/μL | IL-6, pg/mL | 764 | -0.162 | <0.001 | <0.001 |
|  | CRP, mg/L | 699 | -0.137 | <0.001 | <0.001 |
|  | WBC count | 824 | 0.025 | 0.476 | 0.476 |
|  | NLR | 824 | -0.294 | <0.001 | <0.001 |
|  | LMR | 823 | 0.308 | <0.001 | <0.001 |
|  | Albumin, g/L | 835 | 0.129 | <0.001 | <0.001 |
|  | Hemoglobin, g/L | 824 | 0.139 | <0.001 | <0.001 |
|  | Length of stay, days | 844 | -0.037 | 0.285 | 0.304 |

Note. Spearman r values are shown with raw P values and Benjamini-Hochberg FDR-adjusted P values across the 32 correlation tests.

Supplementary Table S4. Comparison of complete-case and incomplete-case patients for multivariable analysis.

| Characteristic | Complete case | Incomplete case | P |
| --- | --- | --- | --- |
| n | 623 | 264 |  |
| Male sex, n (%) | 367 (58.9%) | 145 (54.9%) | 0.272 |
| Disease category, n | Obstruction/perforation acute abdomen: 109;  Acute infection/inflammation: 172;  Benign polyps/diverticular disease: 93;  GI tumors: 108;  Other diseases: 60;  Hernia/abdominal wall diseases: 23;  Benign anorectal diseases: 45;  Stoma-related conditions: 13 | Obstruction/perforation acute abdomen: 25;  Acute infection/inflammation: 86;  Benign polyps/diverticular disease: 43;  GI tumors: 42; Other diseases: 27;  Hernia/abdominal wall diseases: 9;  Benign anorectal diseases: 29;  Stoma-related conditions: 3 | 0.049 |
| Age, years | 59.0 (41.0-71.0); n=623 | 61.0 (43.0-69.0); n=264 | 0.749 |
| Length of stay, days | 7.90 (5.60-14.1); n=623 | 7.10 (5.10-12.0); n=221 | 0.055 |
| T cells, cells/μL | 771 (506-1057); n=623 | 716 (516-969); n=264 | 0.222 |
| NK cells, cells/μL | 139 (87.0-216); n=623 | 143 (93.0-211); n=264 | 0.649 |
| IL-6, pg/mL | 14.4 (5.10-41.3); n=623 | 15.4 (5.00-36.7); n=141 | 0.644 |
| CRP, mg/L | 6.05 (1.67-34.5); n=623 | 1.92 (0.63-26.1); n=76 | 0.004 |
| NLR | 3.62 (2.23-6.56); n=623 | 3.56 (2.11-7.18); n=201 | 0.650 |
| Albumin, g/L | 42.2 (38.5-44.6); n=623 | 42.2 (39.0-45.0); n=212 | 0.272 |

Note. Complete-case patients had all covariates required for multivariable modeling: disease category, sex, age, IL-6, CRP, albumin, NLR, and lymphocyte outcomes. Continuous variables are presented as median (interquartile range); categorical variables are presented as counts or counts with percentages.

Supplementary Table S5a. Linear regression models for log-transformed T-cell and NK-cell counts.

| Outcome | Predictor | Adjusted ratio | 95% CI | P | R² |
| --- | --- | --- | --- | --- | --- |
| T-cell count | Acute infection/inflammation | 1.096 | 0.908-1.323 | 0.338 | 0.326 |
| T-cell count | GI tumors | 1.289 | 1.065-1.560 | 0.009 | 0.326 |
| T-cell count | Obstruction/perforation | 0.867 | 0.714-1.052 | 0.148 | 0.326 |
| T-cell count | Hernia/abdominal wall | 1.087 | 0.830-1.424 | 0.544 | 0.326 |
| T-cell count | Benign polyps/diverticular | 1.328 | 1.096-1.611 | 0.004 | 0.326 |
| T-cell count | Stoma-related | 1.226 | 0.885-1.700 | 0.221 | 0.326 |
| T-cell count | Other diseases | 1.241 | 1.009-1.527 | 0.041 | 0.326 |
| T-cell count | Male vs female | 0.959 | 0.879-1.046 | 0.342 | 0.326 |
| T-cell count | Age, per 10 years | 0.958 | 0.932-0.984 | 0.002 | 0.326 |
| T-cell count | IL-6, log1p transformed | 0.940 | 0.900-0.982 | 0.006 | 0.326 |
| T-cell count | CRP, log1p transformed | 1.019 | 0.980-1.059 | 0.336 | 0.326 |
| T-cell count | Albumin, per 5 g/L | 1.056 | 1.006-1.108 | 0.027 | 0.326 |
| T-cell count | NLR, log1p transformed | 0.712 | 0.659-0.769 | <0.001 | 0.326 |
| NK-cell count | Acute infection/inflammation | 0.983 | 0.780-1.239 | 0.886 | 0.175 |
| NK-cell count | GI tumors | 1.116 | 0.883-1.412 | 0.359 | 0.175 |
| NK-cell count | Obstruction/perforation | 0.810 | 0.638-1.029 | 0.084 | 0.175 |
| NK-cell count | Hernia/abdominal wall | 0.746 | 0.536-1.040 | 0.084 | 0.175 |
| NK-cell count | Benign polyps/diverticular | 0.975 | 0.769-1.235 | 0.832 | 0.175 |
| NK-cell count | Stoma-related | 0.831 | 0.556-1.242 | 0.367 | 0.175 |
| NK-cell count | Other diseases | 0.853 | 0.661-1.100 | 0.220 | 0.175 |
| NK-cell count | Male vs female | 1.055 | 0.948-1.174 | 0.325 | 0.175 |
| NK-cell count | Age, per 10 years | 1.088 | 1.053-1.125 | <0.001 | 0.175 |
| NK-cell count | IL-6, log1p transformed | 1.006 | 0.953-1.062 | 0.830 | 0.175 |
| NK-cell count | CRP, log1p transformed | 0.999 | 0.952-1.047 | 0.960 | 0.175 |
| NK-cell count | Albumin, per 5 g/L | 1.072 | 1.010-1.138 | 0.022 | 0.175 |
| NK-cell count | NLR, log1p transformed | 0.719 | 0.654-0.791 | <0.001 | 0.175 |

Note. All models included 623 complete-case patients. Adjusted ratios are exp(beta) values from linear regression models for log-transformed absolute cell counts. Values below 1 indicate lower expected cell counts. Disease-category estimates use benign anorectal diseases as the reference group. Models were adjusted for disease category, sex, age, log1p(IL-6), log1p(CRP), albumin, and log1p(NLR).

Supplementary Table S5b. Logistic regression models for low T-cell and low NK-cell counts.

| Outcome | Predictor | Adjusted OR | 95% CI | P |
| --- | --- | --- | --- | --- |
| Low T-cell count | Acute infection/inflammation | 1.169 | 0.389-3.513 | 0.782 |
|  | GI tumors | 0.605 | 0.194-1.885 | 0.386 |
|  | Obstruction/perforation | 2.939 | 1.001-8.627 | 0.050 |
|  | Hernia/abdominal wall | 1.471 | 0.358-6.041 | 0.593 |
|  | Benign polyps/diverticular | 0.479 | 0.141-1.621 | 0.237 |
|  | Stoma-related | 1.039 | 0.167-6.455 | 0.967 |
|  | Other diseases | 0.769 | 0.226-2.614 | 0.674 |
|  | Male vs female | 1.244 | 0.799-1.937 | 0.333 |
|  | Age, per 10 years | 1.140 | 0.994-1.307 | 0.061 |
|  | IL-6, log1p transformed | 1.200 | 0.972-1.483 | 0.090 |
|  | CRP, log1p transformed | 0.987 | 0.816-1.194 | 0.891 |
|  | Albumin, per 5 g/L | 0.809 | 0.642-1.018 | 0.070 |
|  | NLR, log1p transformed | 3.077 | 2.075-4.563 | <0.001 |
| Low NK-cell count | Acute infection/inflammation | 0.767 | 0.324-1.813 | 0.545 |
|  | GI tumors | 0.489 | 0.192-1.246 | 0.134 |
|  | Obstruction/perforation | 1.026 | 0.424-2.482 | 0.955 |
|  | Hernia/abdominal wall | 1.654 | 0.507-5.396 | 0.405 |
|  | Benign polyps/diverticular | 0.620 | 0.245-1.573 | 0.314 |
|  | Stoma-related | 0.658 | 0.112-3.853 | 0.643 |
|  | Other diseases | 1.038 | 0.404-2.667 | 0.938 |
|  | Male vs female | 0.866 | 0.582-1.290 | 0.481 |
|  | Age, per 10 years | 0.785 | 0.694-0.888 | <0.001 |
|  | IL-6, log1p transformed | 1.010 | 0.833-1.225 | 0.920 |
|  | CRP, log1p transformed | 1.039 | 0.873-1.236 | 0.666 |
|  | Albumin, per 5 g/L | 0.702 | 0.567-0.869 | 0.001 |
|  | NLR, log1p transformed | 2.112 | 1.487-2.999 | <0.001 |

Note. All models included 623 complete-case patients. Low T-cell count was defined as <=510 cells/μL (events=160), and low NK-cell count was defined as <=89 cells/μL (events=167). Disease-category estimates use benign anorectal diseases as the reference group. Models were adjusted for disease category, sex, age, log1p(IL-6), log1p(CRP), albumin, and log1p(NLR).

**Supplementary Table S7. PCT data availability and elevated PCT summary.**

| Disease category | Total n | PCT measured,  n | Measured, % | PCT, median (IQR) | PCT ≥0.5, n (%) |
| --- | --- | --- | --- | --- | --- |
| Acute infection / inflammation | 258 | 11 | 4.3 | 0.04 (0.03-0.20) | 2 (18.2%) |
| GI tumors | 150 | 36 | 24.0 | 0.14 (0.05-0.47) | 9 (25.0%) |
| Obstruction / perforation acute abdomen | 134 | 24 | 17.9 | 0.77 (0.14-4.94) | 13 (54.2%) |
| Benign anorectal diseases | 74 | 2 | 2.7 | 0.14 (0.08-0.19) | 0 (0.0%) |
| Hernia / abdominal wall diseases | 32 | 2 | 6.3 | 37.84 (18.97-56.72) | 1 (50.0%) |
| Benign polyps / diverticular disease | 136 | 6 | 4.4 | 0.16 (0.04-0.32) | 0 (0.0%) |
| Stoma-related conditions | 16 | 1 | 6.3 | 0.06 | 0 (0.0%) |
| Other diseases | 87 | 6 | 6.9 | 0.42 (0.24-0.57) | 2 (33.3%) |

Note. PCT, procalcitonin; IQR, interquartile range. Percentages for PCT ≥0.5 were calculated among patients with measured PCT. IQR was not calculated for categories with only one measured PCT value.
